# Analysis of forensic autopsy cases associated with epilepsy: Comparison between sudden unexpected death in epilepsy (SUDEP) and not-SUDEP groups

**DOI:** 10.3389/fneur.2022.1077624

**Published:** 2022-12-09

**Authors:** Xian Zhang, Jianhua Zhang, Jinming Wang, Donghua Zou, Zhengdong Li

**Affiliations:** ^1^Department of Cardiology, Kunshan Hospital of Integrated Traditional Chinese and Western Medicine, Jiangsu, China; ^2^Shanghai Key Laboratory of Forensic Medicine, Academy of Forensic Science, Ministry of Justice, Shanghai, China

**Keywords:** sudden unexpected death in epilepsy (SUDEP), forensic pathology, autopsy, post-mortem findings, cause of death

## Abstract

**Background and aims:**

Epilepsy is a common and chronic neurological disorder characterized by seizures that increase the risk of mortality. SUDEP is the most common seizure-related category of death. The study aimed to evaluate the key characteristics between SUDEP and not-SUDEP death cases.

**Methods:**

A retrospective study of forensic autopsy cases from 2002 to 2021, performed by the Academy of Forensic Science (Ministry of Justice, China), identified a total of 31 deaths associated with epilepsy. We compared the different characteristics between individuals who died of SUDEP (SUDEP group) and individuals with epilepsy died suddenly due to unrelated causes (not-SUDEP group).

**Results and conclusions:**

13 cases met the general accepted definition of SUDEP; and 18 cases were classified as not-SUDEP. The mean age of the not-SUDEP group was significantly higher than that of the SUDEP groups (*p* < 0.05) and there were more cases without a clear cause of epilepsy in the SUDEP group than in the not-SUDEP group (*p* < 0.05). Death position differed significantly between the two groups, with more cases dying in the prone position in the SUDEP group (*p* < 0.05). Complete autopsies were performed in 24 of the 31 cases. There were no significant differences in heart, lungs and brain weights, or in ventricular thickness (*p* > 0.05) between the SUDEP and not-SUDEP groups. In addition, compared to the not-SUDEP group, the SUDEP group featured a significantly more cases with coronary lesions (grades 1-3, *p* < 0.05). Neuropathological lesions were identified in 12 of the 13 SUDEP cases (92.3%), cardiac lesions were present in 10 cases (76.9%) and pulmonary edema and pulmonary congestion were present in all cases. The primary cause of death in 13 of the 31 cases was seizure disorder or epilepsy. The primary mechanism of death in SUDEP group was mainly asphyxia while that in the not-SUDEP group was cardiopulmonary failure (*p* < 0.05). Patients in the prone position had a significantly higher risk of asphyxia than those who were not. Here, we investigated the key characteristics between SUDEP and not-SUDEP death cases, which may help to facilitate forensic diagnosis in presumed SUDEP cases.

## Introduction

Epilepsy is a common neurological disease that represents a serious threat to human health, affecting ~70 million people globally (1). The weighted median of the standardized death ratio (SMR) in patients with epilepsy is 2.3 in high-income countries and 2.6 in low-income countries, thus indicating a significantly higher risk of mortality than that in the general population (2). Leading causes of death in epilepsy include the sudden death of unknown causes, status of epilepsy, accidental injury, and suicide (3).

Sudden unexpected death in epilepsy (SUDEP) is defined as a sudden, unexpected death, witnessed or unwitnessed, of a person with epilepsy, for whom a complete postmortem examination does not reveal a specific cause of death (4). This nomenclature was initially defined in 1997 by Annegers (5) and (6). The definition of SUDEP was unified in 2012. According to this definition, SUDEP is defined as a category of death and can be classified into seven subtypes: (1) definite SUDEP, (2) definite SUDEP plus, (3) probable SUDEP/probable SUDEP plus, (4) possible SUDEP, (5) near-SUDEP/near-SUDEP plus, (6) not-SUDEP, and (7) unclassified (7). The new definition showed the extension and refinement of the understanding of the disease. Deaths caused by SUDEP remain a serious public health concern (8). SUDEP is the leading cause of epilepsy-related premature mortality and accounts for 8–17% of deaths among people with epilepsy (9). The calamity of SUDEP preferentially targets young people (10).

Because the diagnosis of SUDEP is made by exclusion of other causes of death, it requires a clinical history of epilepsy, witness statements, details of the scene and circumstances of death, and complete postmortem examinations including toxicology. Therefore, forensic medicine study has also played an important role in SUDEP research, and it provides many important clues to elucidate the mechanism of SUDEP as well (11). The neuropathology and cardiac pathology findings in SUDEP are the main concerns of the autopsy (12–16). However, there are no specific neuropathological and cardiac alterations that can categorically confirm SUDEP. The postmortem examination of SUDEP in the future will be an integration of clinical, pathological, and molecular genetic investigation conducted by both forensic experts and neuropathologists.

Obtaining insight into its pathophysiological mechanisms is a cardinal step toward the prevention and reduction of the incidence of SUDEP. The exact mechanism of SUDEP is unknown but postictal disturbed cardiac or respiratory physiology is implicated (17). Seizures that arise in the cortical region can spread to involve the subcortical regions of the central autonomic network. The ictal activity of the central autonomic network can disrupt the functional connectivity of this network by inhibiting or activating autonomic areas, causing diverse autonomic manifestations, including respiratory and cardiovascular dysfunction (18). Adverse effects of adenosine signaling may also potentiate a fatal outcome in the form of SUDEP by suppressing breathing and arousal in the postictal period (19).

Clinical studies on death, including SUDEP, are challenging. It can hardly be carried out in a trial, but only through patient history reviews, medical records, or surrogate measures such as cardiac and respiratory abnormalities (20). Furthermore, retrospective research is still complicated by the loss of data. Previous studies have either compared individuals who had died of SUDEP to individuals who were alive and had epilepsy (20), or compared individuals who died of SUDEP to individuals who died suddenly due to some unrelated causes without epilepsy. Therefore, in this study, we analyzed forensic autopsy cases associated with epilepsy and compared the characteristics of individuals who died of SUDEP to individuals with epilepsy who died of other causes.

## Materials and methods

### Case data

This study was approved by the Academic Committee of the Academy of Forensic Science (AFS), Ministry of Justice, China. We conducted a retrospective study of all cases investigated by the AFS between 2002 and 2021. The cases, in which the cause of death was listed as a seizure or a clear medical history of epilepsy was confirmed, were sifted out from the AFS autopsy case database. Data for forensic pathological identification, including detailed investigation records, surveillance videos, medical history, and autopsy findings, had been collected by AFS, and all case information was never published in any literature.

### Methods

**Figure 1:**
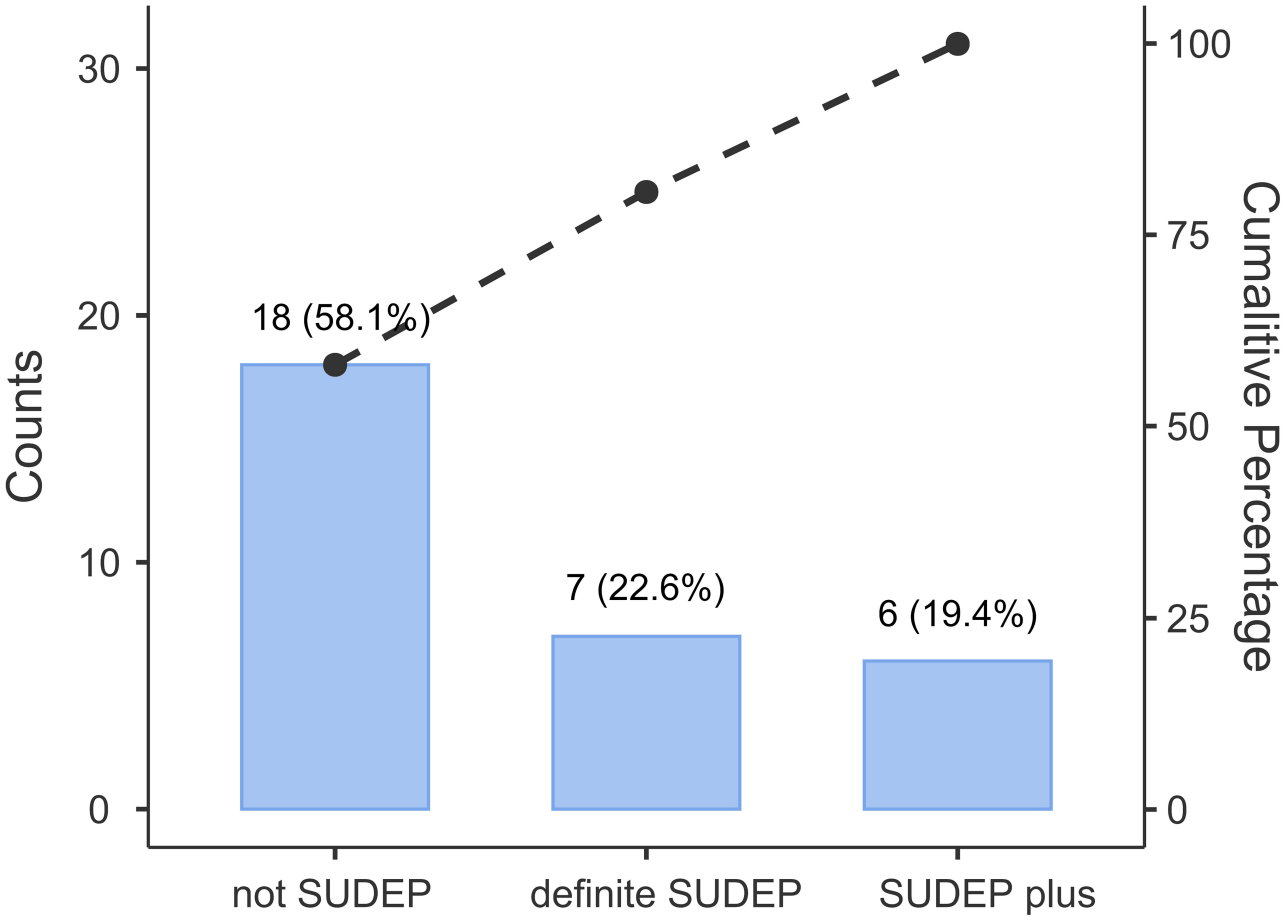
Classification of the collected 31 cases and the respective percentages.

Electronic searches revealed 31 cases for analysis. We reviewed investigation reports and autopsy findings for each of these 31 cases. Cases were then classified according to the latest definition of SUDEP (7). The definitions of each classification are described as below: (1) “definite SUDEP”: a sudden, unexpected, witnessed or unwitnessed, non-traumatic and non-drowning death, occurring in benign circumstances, in an individual with epilepsy, with or without evidence for a seizure and excluding documented status epilepticus (seizure duration ≥30 min or seizures without recovery), in which postmortem examination does not reveal a definite cause of death; (2) “SUDEP plus”: satisfying the definition of definite SUDEP, if a concomitant condition other than epilepsy is identified before or after death, if the death may have been due to the combined effect of both conditions, and if autopsy or direct observations/recordings of terminal event did not prove the concomitant condition to be the cause of death; (3) “probable SUDEP”: same as definite SUDEP but without autopsy; (4) “possible SUDEP”: a competing cause of death is present; (5) “near-SUDEP”: a patient with epilepsy survives resuscitation for more than 1 h after a cardiorespiratory arrest that has no structural cause identified after investigation; (6) “not-SUDEP”: a clear cause of death is known; (7) “unclassified”: not possible to classify. The classification results for the 31 cases are shown in Figure 1. The deceased cases were assigned into two groups: (1) those that met the diagnostic criteria for SUDEP (either definite SUDEP or SUDEP plus) or (2) death unrelated to epilepsy (not-SUDEP). Analysis of etiology was based on the Classification of International League against Epilepsy (ILAE) (21).

Data were then analyzed from several different perspectives: (1) demographic data of the subject, such as age and gender; (2) personal information obtained from records of investigation, including medical history and history of drug or alcohol use; (3) information acquired from the evaluation of the scene and the circumstances of death such as the time of death, the location of death, and the position of the deceased when found; and (4) the cause of death and the mechanism of death.

Cases that met any of the following criteria were excluded to ensure comparability of measurements: (1) The case was an external examination only; (2) The decedent was under 14 years old; and (3) The decedent was decomposing to such a degree as to alter normal organ weights. Finally, the exclusion of 7 cases above left 24 autopsies. All of the 24 cases received a complete autopsy, including histopathological examinations and postmortem toxicological analysis (22). In each autopsy, the heart was dissected 1–2 cm above the aorta and pulmonary trunk. The heart mass was determined by weighing the fresh heart on a metric pan scale after blood and clots were removed from the heart. The epicardial fat was left intact for weighing.

The presence of anti-epileptic drugs (AEDs) was detected by gas chromatography/mass spectrometry (GC/MS). Several AEDs were routinely detected, including carbamazepine, lamotrigine, phenytoin, phenobarbitone, valproic acid, levetiracetam, hydroxycarbazepine, and primidone. For the purpose of forensic identification and this study, therapeutic concentrations were considered as follows: carbamazepine, 4–8 mg/L; lamotrigine, 3–14 mg/L; phenytoin, 10–20 mg/L; phenobarbitone, 10–30 mg/L; valproic acid, 50–100 mg/L; levetiracetam, 10–37 mg/L; zonisamide, 20–30 mg/L; topiramate, 3.4–5.2 mg/L; and hydroxycarbazepine, 12–30 mg/L (23, 24).

### Statistical analyses

Statistical analyses were performed using Jamovi 2 (jamovi.org) (25) and ggstatsplot (26). We checked normal distribution with the Shapiro-Wilk test and verified the homogeneity of variances for each set of data. Normally distributed data are presented as means ± standard error, categorical data are presented as numbers (percentages), and the continuity variables that are not normally distributed are represented by M (Q1, Q3). Statistical testing involved Welch's *t*-test and the Mann-Whitney *U*-test for continuous variables and the chi-square test and Fisher's exact test for categorical variables. To predict whether death position could have exerted an impact on the mechanism of death, we used binomial logistic regression models. Statistical significance difference was defined as *p* < 0.05.

## Results

### Demographic characteristics

A total of 31 cases were classified into three groups according to the latest SUDEP definition; 7 cases (22.6%) met the definition of “definite SUDEP,” 6 cases (19.4%) were classified as “SUDEP plus” group, and the rest 18 cases (58.1%) were defined as “not-SUDEP.” The classification results for the 31 cases are shown in Figure 1.

**Figure 2:**
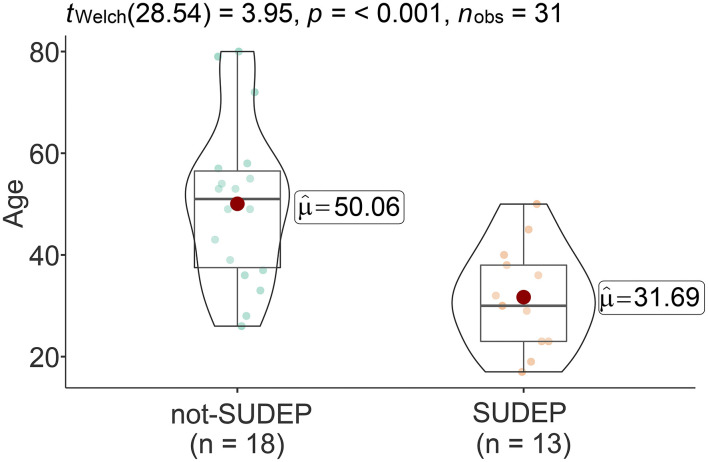
Age comparison between SUDEP and not-SUDEP groups (red dot represents mean value).

Of the 31 cases, 22 were men and 9 were women, and the mean age at death was 42.4 ± 16.3 years (range: 17–80 years). Gender ratios did not vary significantly when compared between the SUDEP and not-SUDEP groups (*p* > 0.05). However, the age of the cases in the not-SUDEP group (50.1 ± 15.9 years) was significantly higher than that of the cases in the SUDEP group (31.7 ± 9.9) (*p* < 0.01). The age comparison results are shown in Figure 2.

### Clinical history and the circumstances of death

In total, 11 cases were associated with epileptic lesions, including intracranial occupancy in three cases and brain trauma in eight cases. The metabolic causes of epilepsy were brain hypoxia and poisoning in four cases; 16 cases had no significant cause of epilepsy. The distribution of cases with or without definite etiology was significantly different when compared between the two groups and more cases in the SUDEP group did not have a definite etiology (*p* < 0.05). Table 1 shows that there were more cases (10/13) with undefined etiology of epilepsy in the SUDEP group than that in the not-SUDEP group (6/18).

**Table 1:** Etiology, death circumstances, and AEDs of the SUDEP cases.

	Not-SUDEP (*N* = 18^*^)	**SUDEP (*N* = 13)**	**χ^2^**	***P***
Etiology of epilepsy			5.74	**0.017**
Not definite [*N* (%)]	6 (33.3)	10 (76.9)		
Definite [*N* (%)]	12 (66.7)	3 (23.1)		
Death location			4.64	0.098
Home	5 (27.8)	6 (46.2)		
Hospital	11 (61.1)	3 (23.1)		
Others	2 (11.1)	4 (30.8)		
Death time	*N* = 16		1.09	0.296
Night	8 (50)	9 (69.2)		
Day	8 (50)	4 (30.8)		
Death position	*N* = 15		7.6	**0.006**
Prone	1 (6.7)	7 (53.8)		
Not prone	14 (93.3)	6 46.2		
Types of AEDs^#^			1.18	0.555
Without	9 (50)	9 (69.2)		
With 1 type	5 (27.8)	2 (15.4)		
With 2 types	4 (22.2)	2 (15.4)		

* Except “death time” and “death position”.

# Anti-epileptic drugs (AEDs). The bold values indicate the values which are less than 0.05, suggesting significant difference.

Investigations indicated that 14 cases (45.2%) died in the hospital or the clinic, while 11 cases (35.5%) died at their residence. Other death locations included a jail cell, a driveway, a massage room, a working place, and in a sewer. The distribution of death locations did not differ significantly when compared between the SUDEP and not-SUDEP groups (*p* > 0.05), as shown in Table 1. By reviewing the case records, a total of 29 cases clearly stated the time of death; two cases did not specify the time of death. The time of death was classified as day and night (daytime was defined as 8:00 a.m. to 8:00 p.m.). No significant difference was detected in terms of the time of death when compared between the SUDEP and not-SUDEP groups (*p* > 0.05), as shown in Table 1. Death positions were analyzed according to case investigations and livor mortis distribution, while 28 cases had a determined death position, and 3 cases of the not-SUDEP group could not determine the death position. Results arising from the SUDEP group differed significantly from the not-SUDEP group. More cases died in the prone position in the SUDEP group (7/13) than that in the not-SUDEP group (1/15) (*p* < 0.05). Details of the death position are shown in Table 1.

### Anti-epileptic therapy, medications, and postmortem toxicological results

Postmortem toxicological analysis revealed that 13 subjects (41.9%) had detectable levels of antiepileptic drugs (AEDs), including valproate (6/13), carbamazepine (6/13), oxcarbazepine (4/13), topiramate (2/13), levetiracetam (1/13), and phenytoin (1/13). Only one case had a supra-therapeutic concentration of carbamazepine; the remaining 12 cases all showed therapeutic concentrations of AEDs. Of the cases taking epilepsy drugs, six took two drugs and seven took one drug. There was no significant difference between the two groups regarding the use of AEDs (*p* > 0.05), as shown in Table 1. One patient had undergone surgery for epilepsy and one other case received electrotherapy. Alcohol consumption was recorded for one case just before death, and traditional Chinese medicine injections were co-administered in another case.

### Pathological findings

In total, 24 of the 31 subjects underwent a complete autopsy, including body surface examination, autopsy, and histopathological examinations.

#### Quantitative comparison of organ weight and ventricular thickness

There were no significant differences between the SUDEP and not-SUDEP groups in terms of the weights of the heart, lungs, and brains, or the ventricular thickness (*p* > 0.05). Further details are shown in Table 2.

**Table 2:** Comparison of organ weight and ventricular thickness of the SUDEP cases.

	**Not-SUDEP (*N* = 11)**	**SUDEP (*N* = 13)**	***T***	***p***
Brain weight (g, $\bar{x}$ ± s)	1287.4 ± 222.2	1390.6 ± 142.9	−1.228	0.242
Heart weight (g, $\bar{x}$ ± s)	317.4 ± 80.5	344.5 ± 78.9	−0.783	0.445
Lung weight (g, $\bar{x}$ ± s)	1410 ± 652.8	1119.5 ± 254.2	1.27	0.234
Left ventricular thickness (cm, $\bar{x}$ ± s)	1.2 ± 0.2	1.2 ± 0.2	−0.422	0.678
Right ventricular thickness (cm, $\bar{x}$ ± s)	0.3 ± 0.05	0.3 ± 0.09	−0.513	0.614

#### Comparison of coronary artery lesions

**Figure 3:**
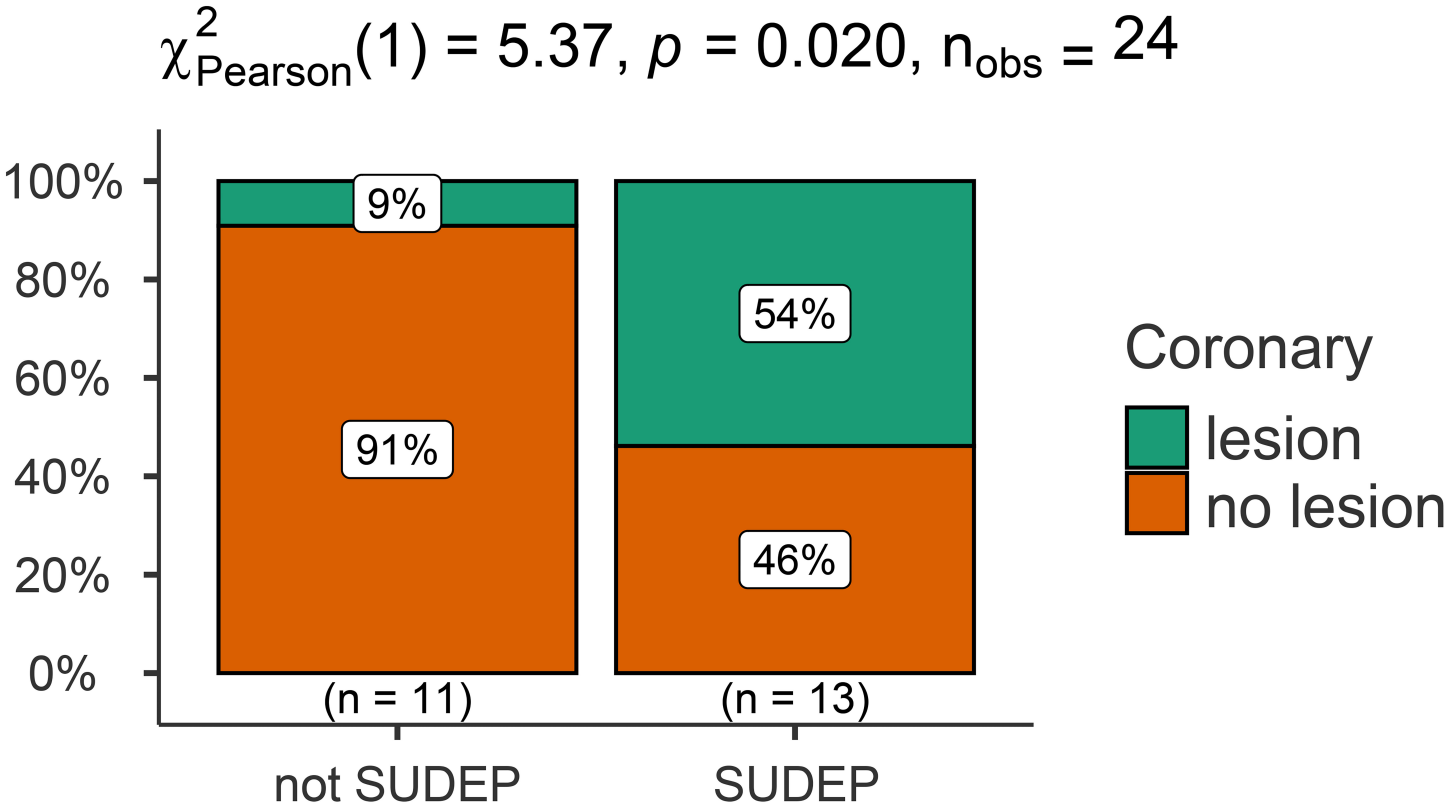
Comparison of the coronary artery lesions between SUDEP and not-SUDEP groups.

Analysis showed that the SUDEP group featured a significantly higher number of cases with coronary lesions (grades 1–3) than that in the not-SUDEP group (*p* < 0.05); further details are shown in Figure 3.

#### Pathological findings in the SUDEP group

**Figure 4:**
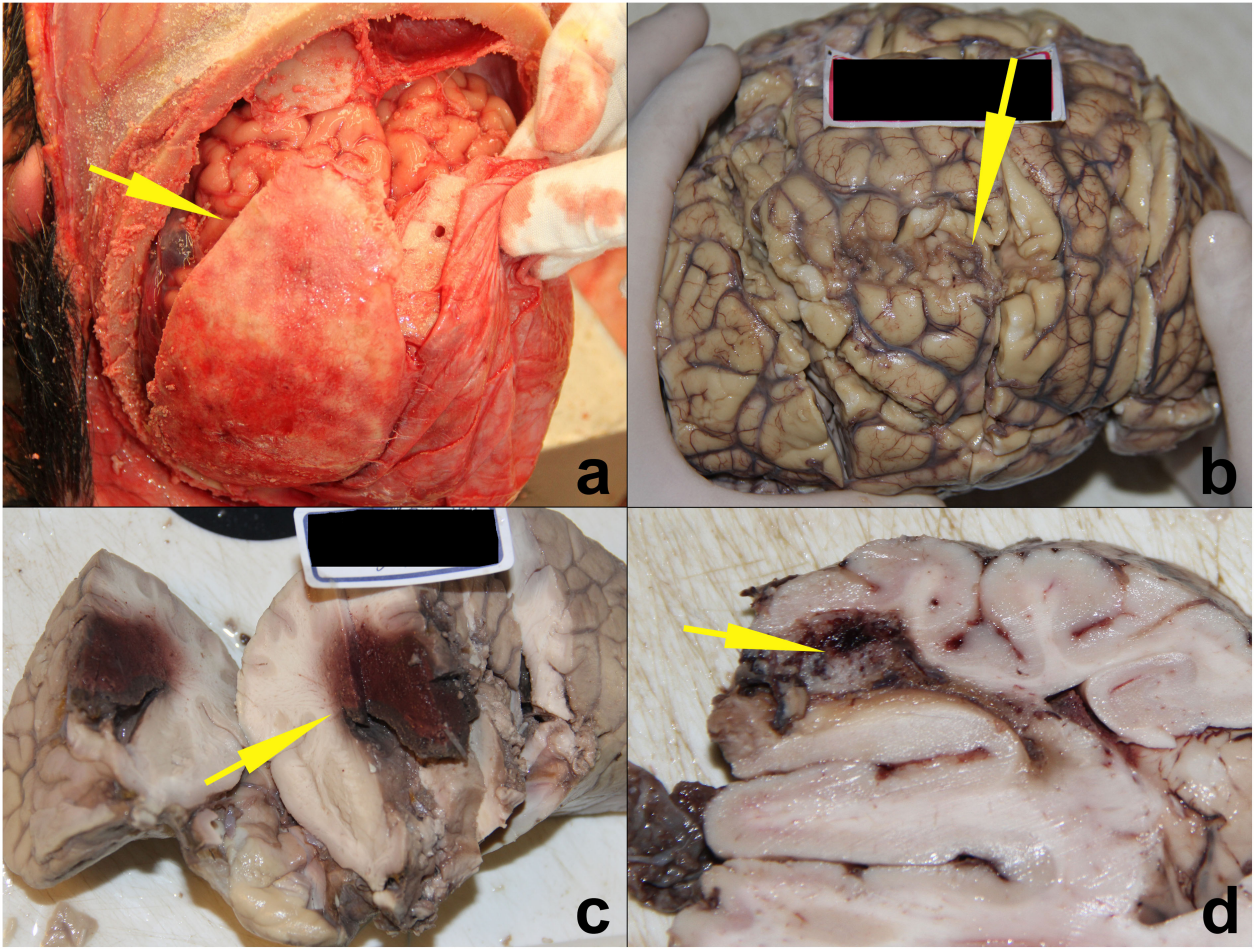
Four SUDEP cases with typical epileptic brain lesions. **(a)** Epilepsy secondary to chronic subdural hematoma ossification. **(b)** Epilepsy secondary to old cerebral contusions. **(c)** Epilepsy secondary to cerebral glioma. **(d)** Epilepsy secondary to vascular malformation. All lesions are indicated by yellow arrows.

Next, we analyzed the main pathological findings of the 13 cases in the SUDEP group. Neuropathological findings were present in 12 cases (92.3%), and four typical epileptic lesions are presented in Figure 4; the remaining cases showed no gross or microscopic abnormalities. Ten cases showed cardiac pathological changes (76.9%), most of which involved subpericardial petechiae; the next most common condition was local myocardial fibrosis. Seven cases (53.8%) had coronary artery atherosclerosis with stenosis degrees of stages I to III. All subjects showed pulmonary congestion and edema. Other pulmonary outcomes included hemorrhage and focal inflammation. Nail cyanosis and other pathological findings were also recorded and shown in Table 3.

**Table 3:** Pathological findings of SUDEP cases.

**Pathological findings**	**Cases (%)** ***N*** **= 13**
Neuropathological findings	
Encephaledema	6 (46.2)
Traumatic lesions	2 (15.4)
Developmental abnormalities	2 (15.4)
Focal encephalomalacia	2 (15.4)
Subarachnoid hemorrhage	1 (7.6)
Vascular malformation	1 (7.6)
Cortical atrophy	1 (7.6)
Tumor	1 (7.6)
Post brain surgery	1 (7.6)
Hippocampi atrophy	1 (7.6)
No pathological findings	1 (7.6)
Cardiac findings	
Subplane-epicardial bleeding	6 (46.2)
Local myocardial fibrosis	4 (30.7)
No pathological findings	3 (23.1)
Coronary arteriosclerosis	
Grade 1	1 (7.6)
Grade 2	3 (23.1)
Grade 3	3 (23.1)
Grade 4	0 (0)
No lesions	6 (46.2)
Pulmonary findings	
Pulmonary congestion	13 (100)
Pulmonary edema	13 (100)
Subpulmonary hemorrhage	4 (30.7)
Pulmonary hemorrhage	2 (15.4)
Focal inflammation	2 (15.4)
Other findings	
Nail cyanosis	12 (92.3)
Palpebral conjunctiva congestion	5 (38.5)
Bronchial foam	5 (38.5)
Tongue between dentitions	4 (30.7)
Intraoral and nasal bleeding	4 (30.7)
Laryngeal edema	1 (7.6)
Facial cyanosis	1 (7.6)
Pale area around the mouth and nose	1 (7.6)

### Causes and mechanisms of death

Table 4 shows the causes and mechanisms of death, as identified by forensic pathologists. Of the 31 cases, SUDEP/epileptic state was listed as the primary cause of death in 14 cases. The mechanism of death was classified as cardiopulmonary failure in 15 cases, asphyxia in eight cases, central nervous system dysfunction in five cases, and sudden cardiac death in three cases.

**Table 4:** The cause and mechanism of death of the SUDEP cases.

**Cause of death**	**Cases (%)** ***N*** **= 31**
SUDEP/epileptic state	13 (41.9)
Trauma/external force	9 (29)
Other medical diseases	4 (12.9)
Toxicosis	3 (9.7)
Accident	2 (6.5)
The death mechanism	
Cardiopulmonary failure	15 (48.4)
Asphyxia	8 (25.8)
Central nervous system dysfunction	5 (16.1)
Sudden cardiac death	3 (9.7)

**Figure 5:**
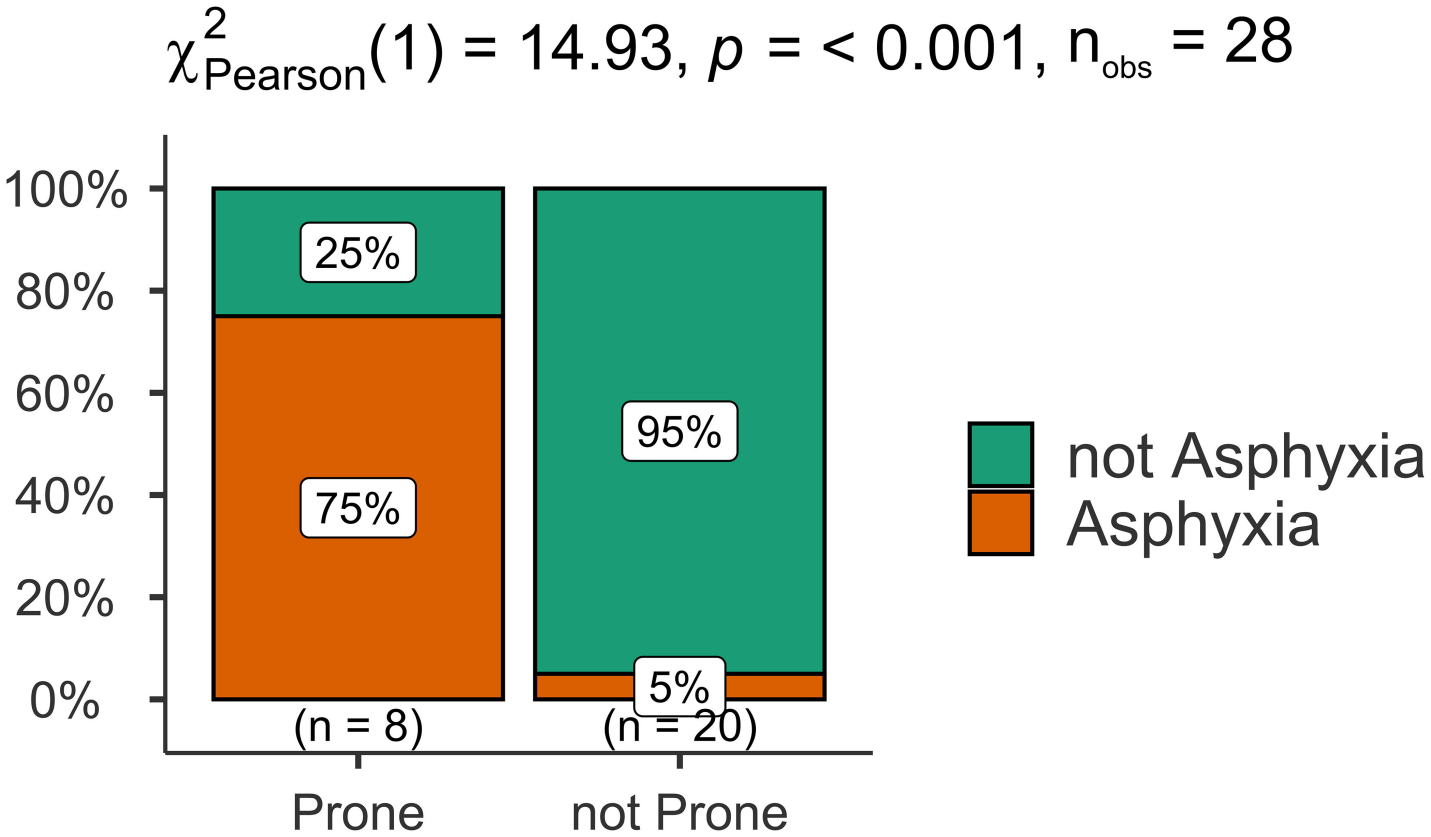
Comparison of the signs of asphyxia between prone and not-prone groups.

Comparative analysis showed that there was a significant difference between the SUDEP and not-SUDEP groups in terms of the mechanisms of death (*p* < 0.05). The main mechanism of death in the SUDEP group was asphyxia; in the not-SUDEP group, the predominant mechanism was a cardiopulmonary failure. Comparative analyses of the mechanism of death are shown in Figure 5.

### Regression analysis of death mechanisms and death positions

**Figure 6:**
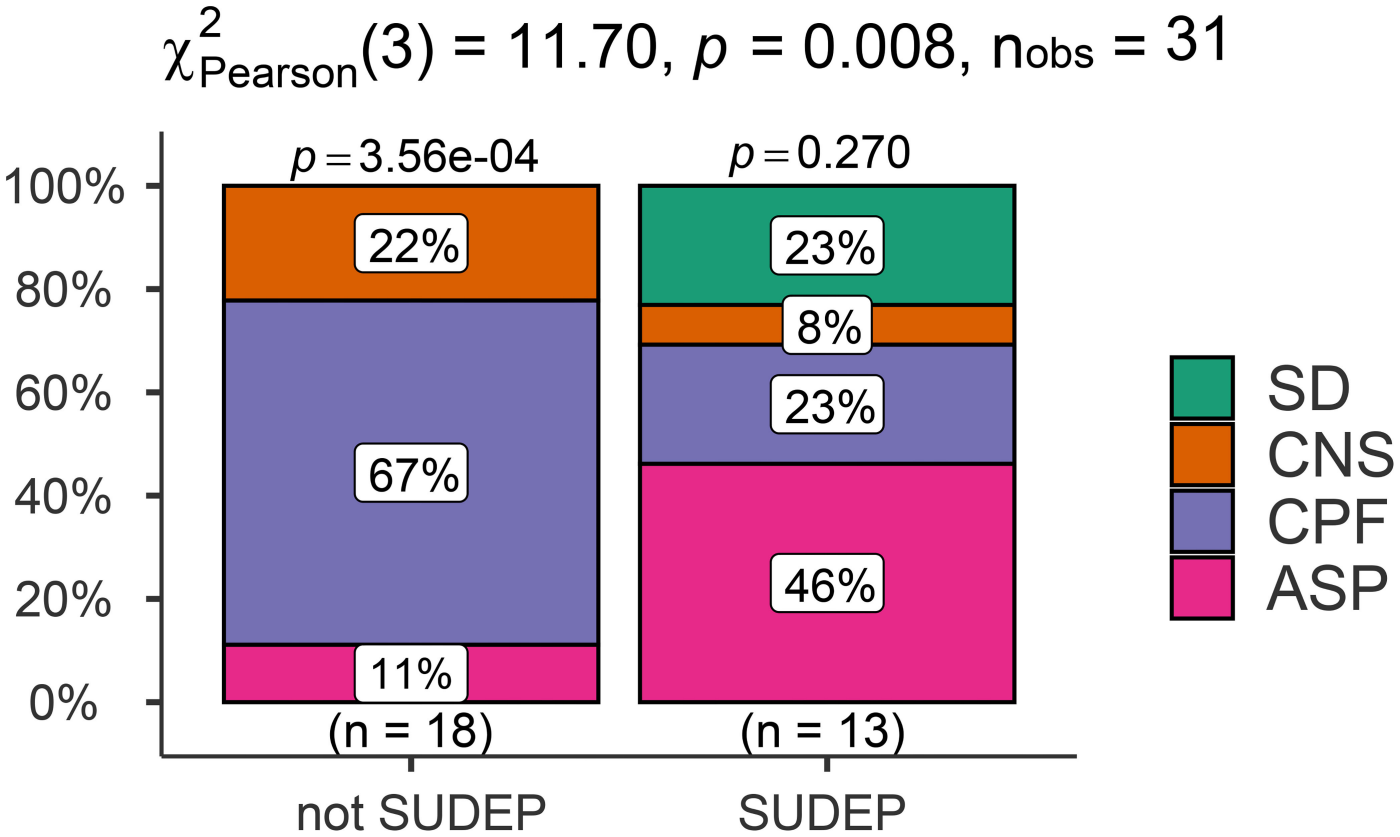
Comparison of the cause of death classification results in the SUDEP and not-SUDEP groups.

Next, we used logistic regression analyses to investigate the effect of death position on the mechanisms of death in the SUDEP group and found that death position had a significant influence on the mechanism of death by SUDEP. Patients in the prone position had a 57-fold higher risk of asphyxia than those in the non-prone position (95% confidence interval [CI]: 4.36–22.26, *p* = 0.002). There was a statistical difference (*p* < 0.05) in the classification of causes of death between the SUDEP and not-SUDEP groups, where the not-SUDEP group died mainly from cardiopulmonary failure (67%), while the SUDEP group died mostly from asphyxia (46%). Further details are shown in Figure 6.

## Discussion

Epilepsy is a common and chronic neurological disorder characterized by seizures (27). Epilepsy can cause death or contribute to the circumstances of death in numerous different ways: status epilepticus, complications following seizure such as aspiration pneumonia, injury or drowning, complications of treatment, or suicide (28).

Sudden unexpected death in epilepsy (SUDEP) is defined as a sudden, unexpected death, witnessed or unwitnessed, of a person with epilepsy, where a complete postmortem examination does not reveal a specific cause of death (47). The definition of SUDEP has been revised over recent years; it is now recognized that SUDEP is actually a general term for a range of diseases. Thus, SUDEP was divided into seven groups by Nashef et al.: (1) definite SUDEP, (2) definite SUDEP plus, (3) probable SUDEP/probable SUDEP plus, (4) possible SUDEP, (5) near-SUDEP/near-SUDEP plus, (6) not-SUDEP, and (7) unclassified (7). The refined classification shows the complex presentation of SUDEP cases. The cases described in this study involved only three groups: definite SUDEP, SUDEP plus, and not-SUDEP groups; our analysis did not involve the other five classifications of SUDEP. It is clear that by definition the “probable SUDEP” and “near SUDEP” groups could not be in the forensic autopsy file records. This result may also be related to the relatively small number of cases in this study, while the small sample size of this study is due to the low autopsy rate in China. Since there is no clear conclusion that shows a different mechanism between the definite “SUDEP” and the “SUDEP plus” groups, we considered the two groups together in the experiment, which is called the SUDEP group, while those deaths with a clear cause of death were included in the not-SUDEP group. We performed a comparative analysis of the demographic profiles, death scenes, medical histories, histopathology results, and mechanisms of death between the SUDEP group and the not-SUDEP group, both of which had epilepsy. Such a grouping is significantly different from the previous studies. Previous studies have either compared individuals who had died of SUDEP to individuals who were alive and had epilepsy (20), or compared individuals who died of SUDEP to individuals without epilepsy who died suddenly due to some unrelated causes, and very few cases have directly compared individuals who died of SUDEP to individuals with epilepsy who died due to other certain causes (29). We considered this comparison to be practically valuable, which revealed another perspective for studying the SUDEP. Because all the death cases are combined with epilepsy, it can eliminate the interference of disease background to the fullest extent, and the comparative results are more convincing in terms of death mechanism.

The risk factors of SUDEP include generalized tonic-clonic seizures, the levels of anti-epileptic drugs (AEDs), frequent seizures, sleep, the prone position, reduced heart rate variability (HRV), and concomitant channelopathies (30–33). However, our analyses identified some new aspects to consider.

Previous studies suggested that nocturnal seizures and the prone position can be related to SUDEP (34, 35). There is a strong association between SUDEP and sleep, with ~70% of SUDEP cases identified during sleep (36). Our analysis of death scenes and medical history found that the SUDEP group differed from the not-SUDEP group in terms of the death position, as there were more deaths in the prone position in the SUDEP group. However, there was no significant difference between the two groups in terms of the time of death and the place of death. It should be noted that the time of death does not fully relate to whether a patient is in a state of sleep; this is because in many cases, the process of death is not witnessed. This may contribute to a divergence in the results of trials. Other case-control investigations of SUDEP postmortems also have shown no evidence for diurnal patterns with respect to SUDEP (37).

The effect of taking anti-epileptic medications on the occurrence of SUDEP may be influenced by the presence or absence of generalized tonic-clonic seizures (GTCS) episodes or sub-therapeutic levels of anti-seizure medications l (35, 38). In the present study, we found no significant difference between the SUDEP and not-SUDEP groups regarding the use of medications when considering postmortem toxicological analysis. Furthermore, we did not identify any cases with sub-therapeutic levels of anti-seizure medications; this may also suggest that the effect of AED use on SUDEP is limited without other qualifying conditions (39).

We also found some interesting phenomena when conducting demographic and etiological analyses. First, in terms of the age at death, we found that cases in the not-SUDEP group were significantly older than those in the SUDEP group (*p* < 0.05). Second, based on medical records, we identified more cases with undefined etiology of epilepsy in the SUDEP group than that in the not-SUDEP group. These findings might suggest that SUDEP may differ from other types of epilepsy in terms of etiology and pathogenesis (40).

A key research focus is whether patients with SUDEP have potentially fatal cardiovascular and cerebral diseases (41). Our statistical analyses found no significant difference in the weights of the heart, lungs, and brains, or the thickness of the ventricles when compared between the SUDEP and not-SUDEP groups (*p* > 0.05). However, the proportion of cases with coronary lesions in the SUDEP group was significantly higher than that in the not-SUDEP group (*p* < 0.05). However, no significant pathological changes of myocardial infarction were detected in these cases with coronary lesions, thus indicating that coronary artery disease is not the main cause of death in SUDEP but may be involved in the nosogenesis of SUDEP. Myocardial ischemia due to coronary heart disease may induce abnormal ECG activity rather than myocardial infarction which could then participate in the development of SUDEP. This is potentially supported by the findings that T-wave alternans is considered a potential biomarker for SUDEP (42–44).

Analysis of the mechanism of death is an important aspect of forensic pathology practice and is also the focus of SUDEP (43). The mechanism of SUDEP involves neuropeptidergic, serotonergic, and adenosine systems, as well as alterations of the ventrolateral medulla, amygdala, hippocampus, and central autonomic regions, orchestrating autonomic dysfunction (45). In the present study, pooled analysis of 31 cases of SUDEP identified the mechanisms of death in epileptic patients as asphyxia, cardiopulmonary dysfunction, central nervous system dysfunction, and sudden cardiac death. Our results showed that the mechanisms of death differed between the definite SUDEP and not-SUDEP groups. Most of the cases in the definite SUDEP group showed signs of asphyxia, such as nail cyanosis, hemorrhage in the bulbar conjunctiva, pulmonary pleura, and the sub-epicardium. To explain this phenomenon, we performed regression analysis on the death position most likely to cause asphyxia and found a strong correlation between death position and asphyxia, thus suggesting that the prone position was the main factor associated with asphyxia in patients with SUDEP. Ictal and postical effects on autonomic function and accidental asphyxia are commonly considered potential factors of SUDEP (18). As such, SUDEP may share mechanisms similar to sudden infant death syndrome (31).

However, due to the small number of cases, the conclusions of this study may need to be verified by further studies with larger sample sizes. In addition, due to the lack of background data, and the lack of awareness of SUDEP in many cases during the autopsy, there may be a lack of intensive and detailed pathological examinations, such as immunohistochemistry studies for mild malformations of cortical development (MCD) (46). Thus, more detailed results concerning the mechanisms of death could not be obtained.

## Conclusion

Based on the forensic death cases, we conducted a statistical analysis of deaths in patients with epilepsy, focusing specifically on the comparative analysis of individuals who died of SUDEP (SUDEP group) to individuals with epilepsy who died due to other certain causes (not-SUDEP group). Significant differences were founded between the two groups: compared to the not-SUDEP group, the SUDEP group was younger in age; the SUDEP group has more cases with uncertain etiology of epilepsy, prone death position, and coronary lesions. The primary mechanism of death in the SUDEP group was mainly asphyxia while that in the not-SUDEP group was a cardiopulmonary failure. Additionally, asphyxia in the SUDEP group correlates significantly with the prone position. This will provide new ideas and directions for further research and forensic identification on SUDEP.

## Data availability statement

The raw data supporting the conclusions of this article will be made available by the authors.

## Ethics statement

The studies involving human participants were reviewed and approved by Academic Committee of the Academy of Forensic Science (AFS), Ministry of Justice, PR China. Written informed consent to participate in this study was provided by the participants' legal guardian/next of kin.

## Author contributions

XZ and ZL conceptualized the study. ZL responsible for the methodology. XZ and JZ responsible for the formal analysis. XZ wrote the original draft. DZ supervised the research. JW provided administration services. DZ and ZL acquired the funding. All authors read and agreed to the published version of the manuscript.
